# Two Paradoxostomatidae (Ostracoda) species from South Korea with a key to genera of the family

**DOI:** 10.3897/zookeys.943.52938

**Published:** 2020-06-22

**Authors:** Hyunsu Yoo, Van Anh Le Thi, Ivana Karanovic

**Affiliations:** 1 Marine Environmental Research and Information Laboratory (MERIL), 17, Gosan-ro, 148 beon-gil, Gunpo-si, Gyoenggi-do, 15180, South Korea Marine Environmental Research and Information Laboratory Seoul South Korea; 2 Department of Life Science, College of Natural Sciences, Hanyang University, Seoul, 04763, South Korea Hanyang University Seoul South Korea; 3 Institute for Marine and Antarctic Studies, University of Tasmania, Hobart, Tasmania, Australia University of Tasmania Tasmania Australia

**Keywords:** Biodiversity, Cytheroisinae, East Asia, taxonomy

## Abstract

*Cytherois gajinensis***sp. nov.** is described and *Violacytherois sargassicola* (Hiruta, 1976) is redescribed. The species have been collected from littoral and interstitial waters in South Korea. They belong to Cytheroisinae, one of the three Paradoxostomatidae subfamilies. Both species are the first taxonomic records of the subfamily in Korea. Taxonomic keys to the living Paradoxostomatidae genera are provided in an attempt to clarify the position of some of the currently included genera as well as a key to East Asian Cytheroisinae species in order to facilitate further biodiversity research in the region.

## Introduction

The family Paradoxostomatidae comprises ostracods with a fragile, elongated, and laterally compressed carapace (Cohen et al. 2007). They are mostly algal dwellers (Insafitri and Kamiya 2014), but several genera have been reported living commensally (see Tanaka and Arai 2017; Tanaka and Hayashi 2019). Paradoxostomatidae is the only ostracod taxon in which the upper and lower lips are fused into a suctorial disk, functioning as piercing and/or sucking organs (Athersuch et al. 1989). This enables animals to stick to the surface of seaweeds and, depending on the size of mouthparts, they specialize to different types of algae (Insafitri and Kamiya 2014).

According to the World Ostracoda Database (Brandão et al. 2020), the family comprises 25 genera, but the list does not include recently described commensal genus, *Chelonocytherois* Tanaka & Hayashi, 2019. The family’s main distinguishing character is a very reduced maxillular palp, mandibular palp, and mandibular coxa. Based on the level of these reductions the family is divided into three subfamilies: Cytheroisinae, Paracytheroisinae, and Paradoxostomatinae (see Schornikov 1993). Paracytheroisinae comprises only one genus, *Paracytherois* Müller, 1894 characterized by a long styliform mandibular coxa and its palp reduced into a long, whip-like seta (see Athersuch et al. 1989). Members of Cytheroisinae are on the opposite end of the reduction of mandibula, with more robust coxa and the palp consisting of at least two segments and several setae. This subfamily includes the following genera: *Cytherois* Müller, 1884; *Chelonocytherois*, *Flabellicytherois* Schornikov, 1993, and *Violacytherois* Schornikov, 1993. Furthermore, *Cytherois* is subdivided into two subgenera, the nominal and *Orientocytherois* Schornikov, 1993. Representatives of Paradoxostomatinae have mandibular palp similar to Cytheroisinae, while the coxa is similar to Paracytheroisinae. Paradoxostomatinae includes the rest of 20 Paradoxostomatidae genera, although position of many is doubtful (see discussion). Its most diverse genus, *Paradoxostoma* Fischer, 1855, has been revised several times, and most recently by Schornikov and Keyser (2004) who erected five genera to mirror morphological diversity of this taxon.

Although South Korean cytheroids are poorly studied in general (see Karanovic et al. 2017), with 52 species described/reported so far (Yoo et al. 2019), Paradoxostomatidae, and in particular *Paradoxostoma*, with eleven species, is by far the best studied genus from this country. In addition to those 52 named species, Lee et al. (2000) list another 400-plus cytheroids; however, they are mostly unidentified as their research was related to studying water pollution, rather than biodiversity. Their list includes 25 unnamed *Cytherois* species and one provisionally identified, C. cf. megapoda Schornikov, 1993.

Here we report on two Cytheroisinae species from South Korea. One is a new species of *Cytherois* and the other is *Violacytherois sargassicola* (Hiruta, 1976). *Cytherois* is by far the most diverse genus in the subfamily comprising about 60 species (see Brandão et al. 2020). Of those, more than 1/3 are known only after the shell, either because they are subfossil/fossil species, or because of an insufficient description. The following species have been reported or described from East Asia (species known only after their shells are marked with asterisk): *C. asamushiensis* Ishizaki, 1971*; *C. decorata* Okubo, 1980; *C. ikeyai* Nakao & Tsukagoshi, 2002; *C. leizhouensis* Gou and Huang in Gou, Zheng & Huang, 1983*; *C. megapoda* Schornikov, 1993; *C. marginalis* Hu, 1984*; *C. tosaensis* (Ishizaki, 1968); *C. uranouchiensis* Ishizaki, 1968*; *C. wangchieni* Hu & Tao, 2008*; and *C. zosterae* (Schornikov, 1975). *Cytherois asamushiensis*, *C. decorata*, *C. ikeyai*, *C. tosaensis*, *C. uranouchiensis*, and *C. zosterae* are all known from Japan (Ishizaki 1968, 1971; Okubo 1980; Schornikov 1975; Nakao and Tsukagoshi 2002); *C. leizhouensis* was described from China (Gou et al. 1983); *C. marginalis* and *C. wangchieni* from Taiwan (Hu 1984; Hu and Tao 2008); and *C. megapoda* from Russia (Schornikov 1993).

Both *Flabellicytherois* and *Chelonocytherois* are monospecific and endemic to East Asia (Okubo 1980; Schornikov 1993; Tanaka and Hayashi 2019). *Violacytherois sargassicola* was originally described from Hokkaido Island (Hiruta 1976) and later found in the Russian Far East (Schornikov 1993). It is one of the only three species known so far, all endemic to East Asia as well.

Beside the description and redescription of two Cytheroisinae species, we also provide a key to all living genera of Paradoxostomatidae and living East Asian species of Cytheroisinae.

## Materials and methods

Samples were collected by scientific scuba diving (Pardo 2014) and by algal rinsing (hand-net mesh size is 62 um), as described by Giere (2009). Samples were fixed in 99% ethanol on site. Sorting was done under a stereomicroscope (Olympus SZX12) in the Laboratory at Hanyang University. Specimens were dissected, and soft parts mounted on slides in CMC-10 Mounting Media (Masters Company, Inc.), while carapaces were kept on the micro-palaeotological slides. All drawings were prepared using a drawing tube, attached to the Olympus BX51 microscope. For observations under the scanning electron microscope (SEM), carapaces were coated with platinum. SEM photographs were taken at Eulji University with the Hitachi S-4700 electron microscope. All specimens are deposited in the invertebrate collection of the National Institute of the Biological Resources (NIBR) in South Korea.

Abbreviations used in text and figures:

**A1** Antennula;

**A2** Antenna;

**GF** Genital field;

**H** Height;

**Hp** Hemipenis;

**L** Length;

**LV** Left valve;

**L5-7** Leg 5-7;

**Md** Mandibula;

**Mxl** Maxillula;

**RV** Right valve.

## Results

### Systematics

#### Order Podocopida Sars, 1866

**Superfamily Cytheroidea Baird, 1850**

**Family Paradoxostomatidae Brady & Norman, 1889**

**Genus *Cytherois* Müller, 1884**

##### Cytherois gajinensis sp. nov.

Taxon classificationAnimaliaPodocopidaParadoxostomatidae

96C43FCF-F045-562A-8310-2D85D8CF702A

http://zoobank.org/75B1179A-7333-4570-9178-570AA30B4106

Figures 1 , 2 , 3

###### Material examined.

***Holotype***, male, dissected on one slide (NIBRIV0000813439) and shell on micropalaeontological slide; allotype, female, dissected on one slide and shell on micropalaeontological slide; ***paratypes***: two males dissected on each slides and shell on micropalaeontological slides, one female dissected on one slide and shell on micropalaeontological slide and five specimens kept in a 2 ml vial.

**Type locality.** South Korea, Gangwon-do, Goseong-gun, Jugwang-myeon, Gajin-ri; 38°18.16'N, 128°34.36'E, 25 m, sandy bottom; 29 Aug. 2016, collected by Raehyuk Jeong and Wonchoel Lee.

**Etymology.** The species is named after the beach from where it was collected.

###### Description of male.

**Figure 1.**
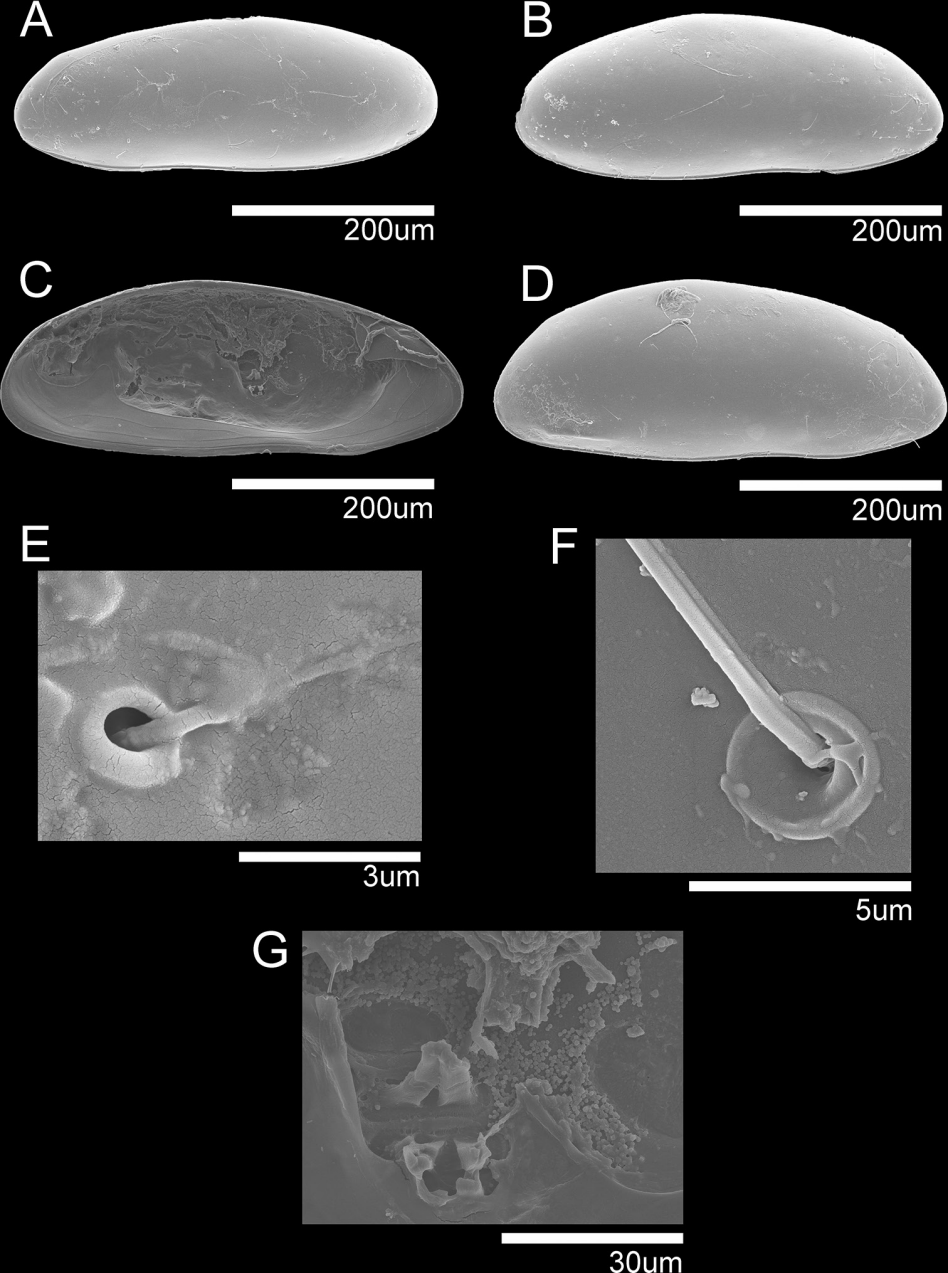
SEM photographs of *Cytherois gajinensis* sp. nov. **A–C, E–G** male **D** female: **A**LV external view (holotype) **B**RV, external view (paratype) **C**LV, internal view (paratype) **D**RV, external view (allotype) **E, F** surface pores (holotype) **G** muscular scar print (paratype).

**Figure 2.**
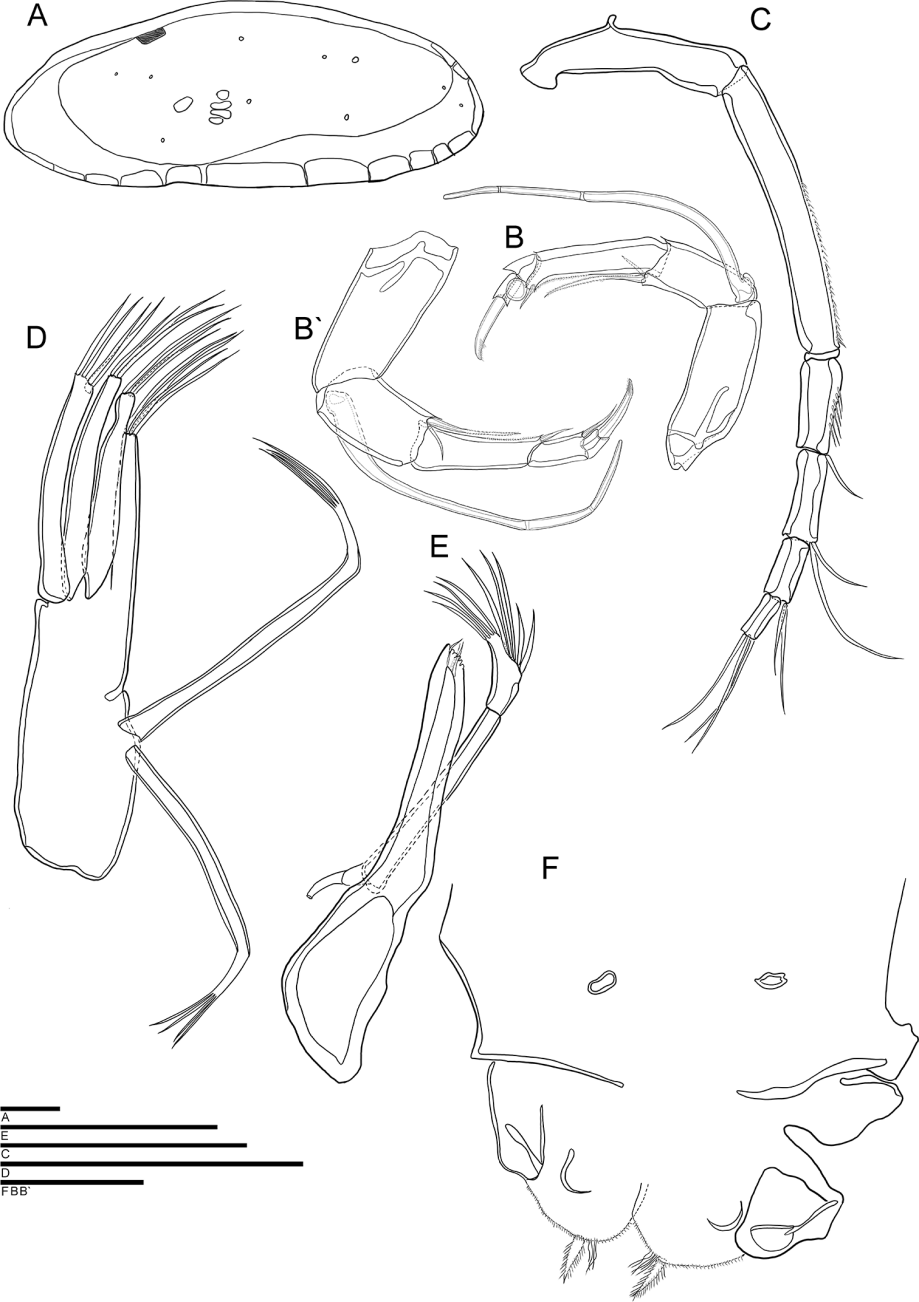
*Cytherois gajinensis* sp. nov. **A–E** male (holotype) **F** female (allotype): **A**RV internal view **B**A2**C**A1**D**Mxl**E**Md**F, B'**GF. All scale bars: 50 µm.

***Carapace*** (Figs 1A–C, E–G, 2A). Relatively small, with L approximately 422 µm, H approximately 154 µm. LV overlapping RV. Carapace elongated ellipsoidal in lateral view (Fig. 1A). Dorsal margin slightly arched, antero-dorsal and postero-dorsal margins evenly curved, ventral margin slightly sinusoid around mouth region. Anterior and posterior margins rounded, with anterior margin being slightly narrower than posterior one. Greatest H situated slightly behind the middle. Eye present. Surface of the carapace smooth with few simple type setae distributed (Fig. 1E, F). Marginal pore canals noticeable along ventral and posterior margins (Fig. 2A), relatively short and not branched. Inner lamella equally wide anteriorly and posteriorly. Muscular scar imprints consisting of a row of four vertical scars and one frontal scar present (Figs 1G, 2A). Hinge adont (Fig. 1C).

***A1*** (Fig. 2C). Six-segmented. First segment without setulae and setae. Second segment with setule along anterior to distal margin. Third segment with visible setulae along anterior to distal margin and one bare seta on antero-distal margin, not reaching end of fourth segment. Fourth segment with two bare setae on antero-distally, one reaching end of fifth segment and another twice longer than the fifth segment. Fifth segment with two bare setae on antero-distal part, one 1.5 times longer than terminal segment and the other twice as long as terminal segment. Terminal segment with three long bare setae on distal margin, almost 2.5 times longer than terminal segment. L ratio between six segments 4.1: 5.6: 1.7: 1.7: 1.36: 1.

***A2*** (Fig. 2B). Five-segmented. Exopod transformed into three-segmented spinneret seta. First endopodal segment without setulae and seta. Second segment with two setae postero-distally: one plumose, seta reaching end of third segment, another bare, reaching 1/3 the third segment. Third segment with setule along posterior to distal margin, and one short, strong, bare seta postero-distally reaching distal end of terminal segment. Penultimate segment with seta transformed into sucking organ. Terminal segment very short and carrying only one strong claw on distal margin. L ratio between five distal segments: 6: 3.1: 4.3: 1: 1.

***Md*** (Fig. 2E). Coxa with six tiny teeth and three thin, bare, setae on distal margin. Exopod with one seta; endopod 2-segmented. First endopodal segment elongated but not carrying any seta. Second segment with nine setae, five of which arise from central margin, four from distal margin. First segment almost four times longer than second segment.

***Mxl*** (Fig. 2D). Palp 1-segmented carrying four bare setae on distal margin, all setae almost half as long as the palp. Two long mop-shaped setae (“aberrant setae”) present at the distal end of vibratory plate. Masticatory process with three endites, first and second endites each with three bare setae, third endite with four bare setae on distal margin.

**Figure 3.**
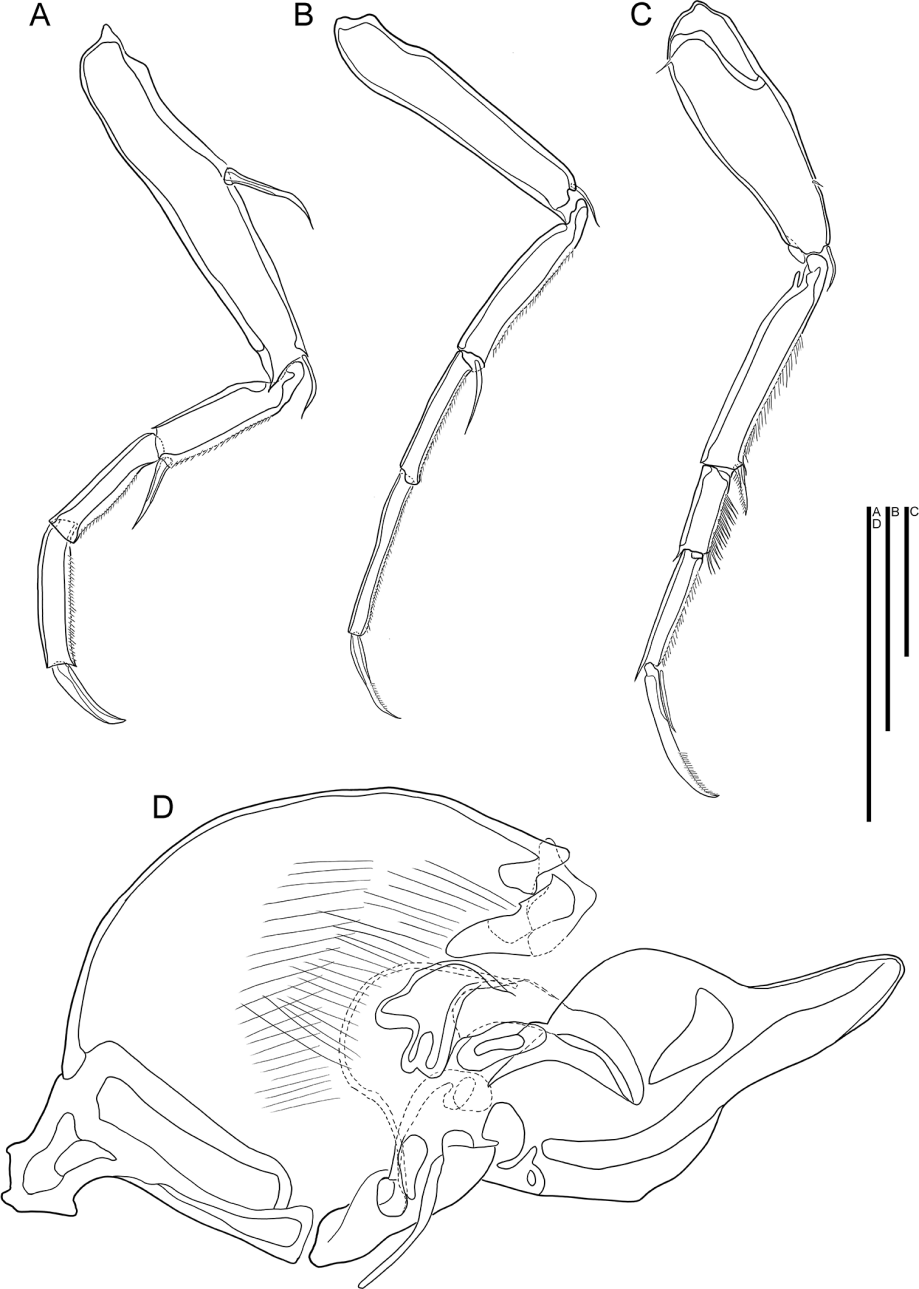
*Cytherois gajinensis* sp. nov. male (holotype) **A** L5 **B** L6 **C** L7 **D**Hp. All scale bars: 50 µm.

***L5*** (Fig. 3A). Four-segmented. First segment with two bare setae, one on antero-medial margin, not reaching end of first segment, and another on antero-distally, reaching 1/3 of second segment. Second segment with one bare seta antero-distally, not reaching end of third segment. Penultimate segment without any seta. Terminal segment with one claw like seta on distal margin. Last three segments with setulae along posterior to distal margin. L ratio between four segments 2.7: 1.24: 1: 1.06.

***L6*** (Fig. 3B). Four-segmented. First segment with one bare seta antero-distally, reaching 1/4 of second segment. Second segment with one bare seta antero-distally, reaching half of third segment. Following segment without any setae. Terminal segment with one claw like seta on distal margin. Last three segments with setulae along posterior to distal margin. L ratio between four segments 2.2: 1.4: 1: 1.3. In comparison to L5, L6 has more elongated segments.

***L7*** (Fig. 3C). Four-segmented. First segment with tiny setule postero-proximally and, antero-medially, and one bare seta on antero-distal margin, reaching 1/4 of second segment. Second segment with one plumose seta on antero-distal margin reaching almost half length of the third segment. Third segment with long, almost spine-like setulae along anterior to distal margin. Terminal segment with one strong claw and one bare seta on distal margin, almost half as long as same segment. Second and terminal segment with setulae along posterior to distal margin. L ratio between four segments 2.9: 2.5: 1: 1.25. Segments of L7 are more elongated than on L5, but less than on L6.

***Hp*** (Fig. 3D). Basal plate ovate. Distally Hp carrying a large lobe in a shape of eagle beak, dorsally to which a much smaller lobe-like process with triangular, but dull tip present.

###### Description of female.

***Carapace*** (Fig. 1D). Slightly larger than males. L approximately 451 µm, H approximately 182 µm. Shape and all other morphological features similar to male.

***A2*** (Fig. 2B'). Penultimate segment with one seta instead of sucking organ, and same segment longer than in male. L ratio between five distal segments of female A2. 9: 5.7: 6.5: 3.5: 1.

***GF*** (Fig. 2F). Basal part rectangular. Two caudal rami present and long setulae cover the surface. End of the body seta not observed.

All other appendages same as in male.

#### Genus *Violacytherois* Schornikov, 1993

##### Violacytherois sargassicola

Taxon classificationAnimaliaPodocopidaParadoxostomatidae

(Hiruta, 1976)

42C9C779-43DD-5C04-8D87-A482956D0FD9

Figures 4 , 5 , 6

Cytherois sargassicola Hiruta, 1976: 24, figs 1–3.
Violacytherois sargassicola (Hiruta): Schornikov, 1993: 181, figs 7, 8; pl II, figs 7–10.

###### Material examined.

Male, dissected on one slide (NIBRIV0000813440) and shell on micropalaeontological slide; Female, dissected on one slide and shell was broken; two males dissected on one slide each, shell broken; one female dissected on one slide, shell broken; one juvenile dissected on one slide; shell on micropalaeontological slide and 12 specimens kept in 2 ml vial in alcohol.

###### Locality.

South Korea, Gyeongsangnam-do, Goseong-gun, Donghae-myeon, Dongdong beach; 34°59.63'N, 128°26.02'E, 0.5 m depth; 04 Apr. 2012; collected by Tomislav Karanovic and Ivana Karanovic.

###### Description of female.

**Figure 4.**
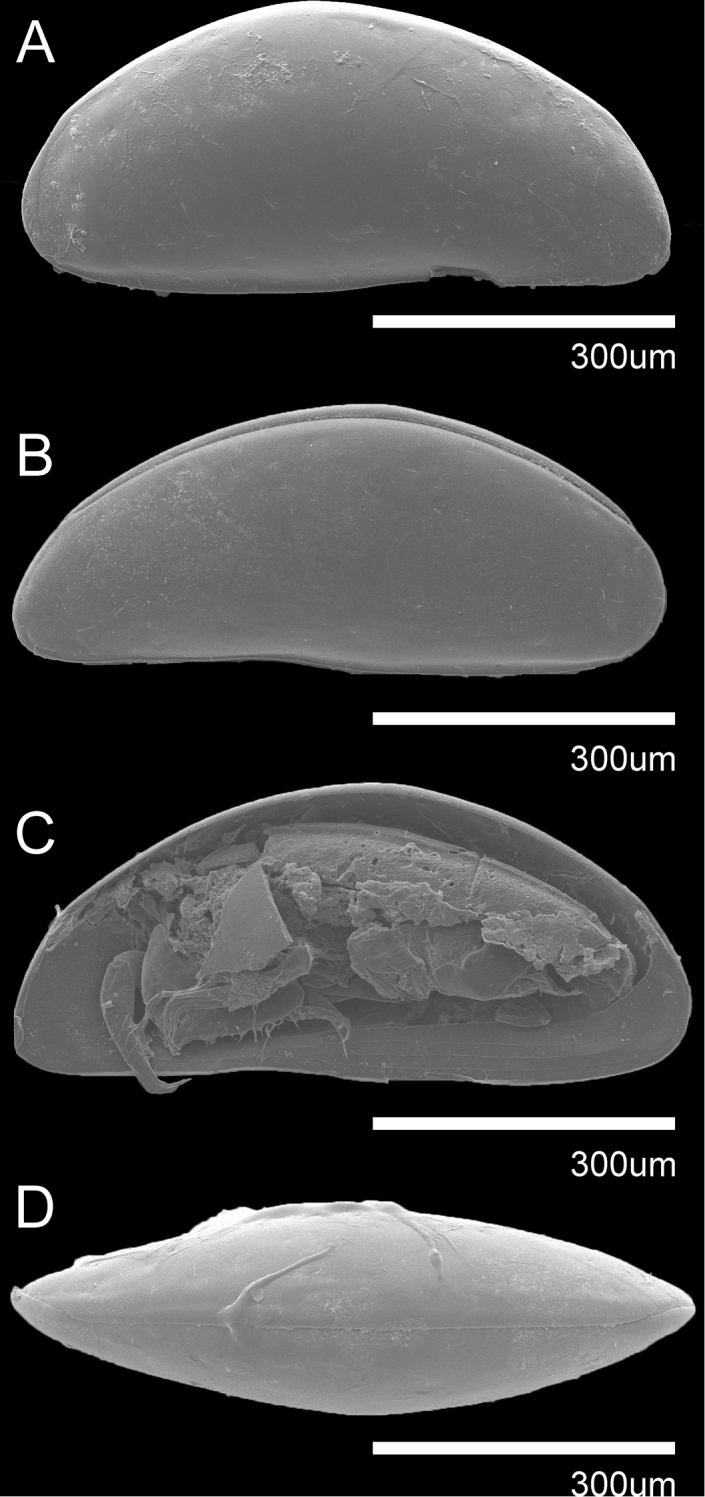
SEM photographs of *Violacytherois sargassicola* (Hiruta, 1976) **A, D** female **B, C** male: **A**RV external view **B**LV external view **C**RV internal view with soft parts **D** dorsal view.

**Figure 5.**
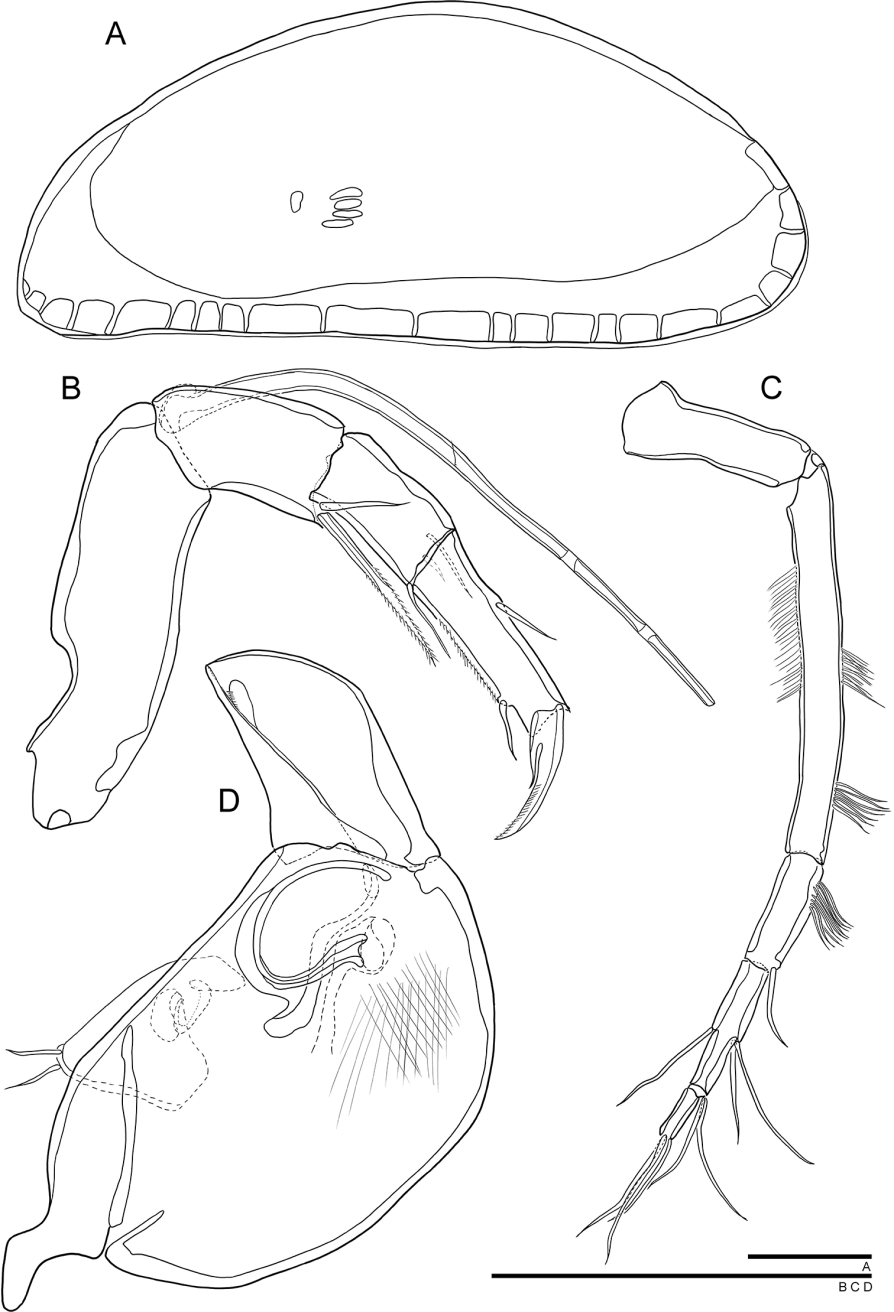
*Violacytherois sargassicola* (Hiruta, 1976) **A** female **B–D** male: **A**RV internal view **B**A2**C**A1**D**Hp. All scale bars: 100 µm.

***Carapace*** (Figs 4A, 5A). L approximately 647 µm, H approximately 295 µm. Carapace ellipsoidal in lateral view (Figs 4A, 5A). Dorsal margin arched, antero-dorsal margin slightly curved, ventral margin almost straight with weak curve point near the middle, the greatest H which is situated slightly behind the middle. Eye absent. LV overlapping RV on anterior and posterior margin, conversely RV overlapping LV on dorsal margin (Fig. 4D). Surface of the carapace smooth with few simple setae. Pore canals sparse, straight and distributed along the margin (Fig. 5A) not branched. Inner lamella wide at anterior margin and increasingly wider ventral, while almost the same with posteriorly. Muscular scar imprints consisting of a row of four vertical scars and one frontal scar present (Fig. 5A). Hinge adont (Fig. 4C).

***A1 and A2*** same as in male (see description below).

**Figure 6.**
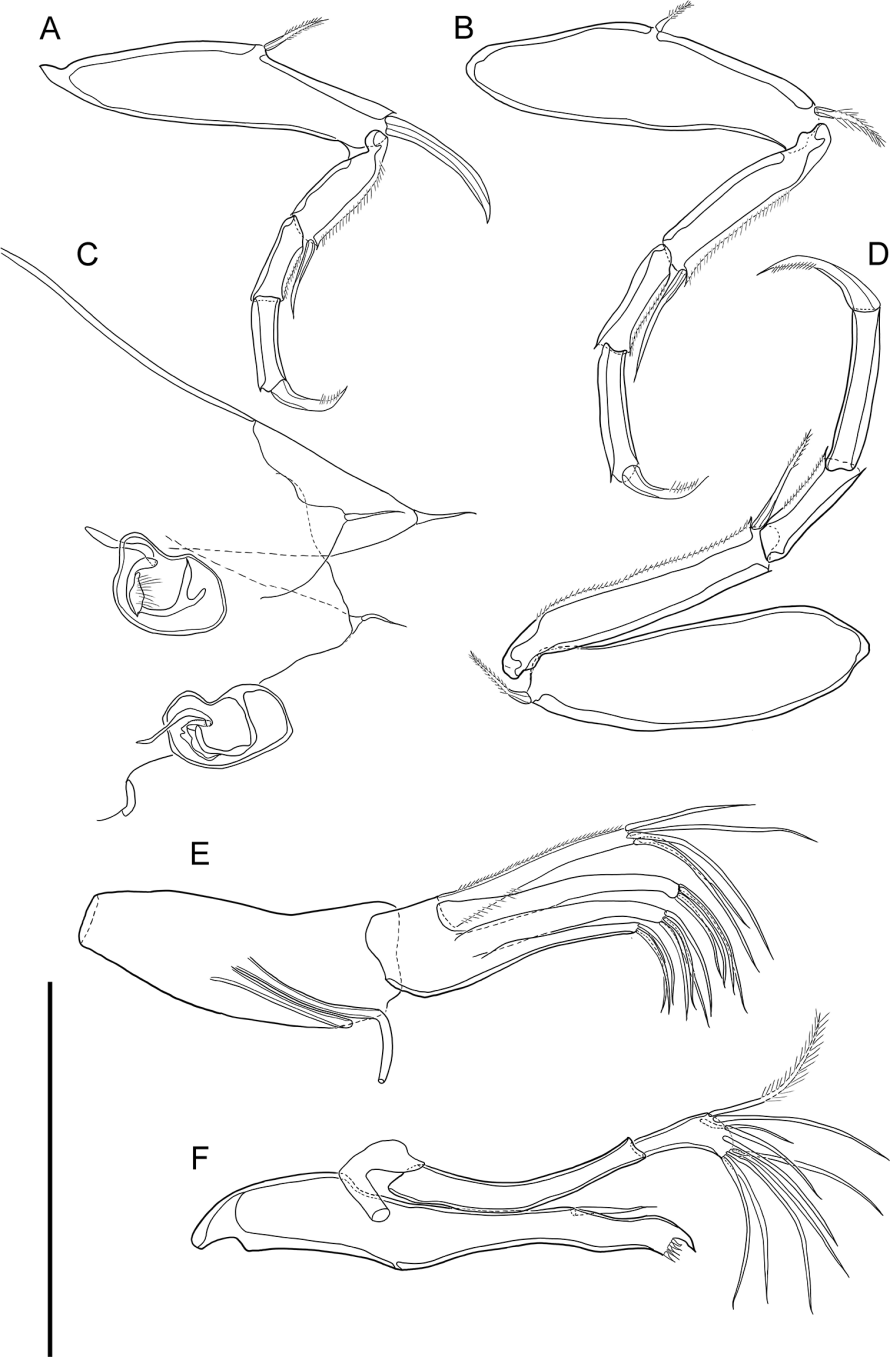
*Violacytherois sargassicola* (Hiruta, 1976): Female **A** L5 **B** L6 **C**GF**D** L7 **E**Mxl**F**Md. All scale bars: 100 µm.

***Md*** (Fig. 6F). Coxa with five small teeth and one strong tooth on distal margin, one bare seta antero-medially not reaching end of the antero-distal margin. Palp with two-segmented endopodite and exopodite carrying one bare seta (broken). First endopodal segment without any seta, almost three times as long as second segment. Second segment with ten setae, one plumose and one bare seta on antero-distally, eight bare setae on distal margin.

***Mxl*** (Fig. 6E). Palp present with five bare setae on distal margin almost same length as the palp segment, setulae present along anterior to distal margin and posterior-proximally. Two long setae at the middle of vibratory plate (aberrant setae). Masticatory process with three endites, first and second endites each with four bare setae almost half length of palp segment, third endite with three bare setae almost 1/3 length of palp segment.

***L5*** (Fig. 6A). Four-segmented. First segment with one plumose seta antero-medially not reaching end of the same segment, one claw-like seta on antero-distal margin. Second segment with setulae along anterior to distal margin, one bare seta antero-distally, reaching 1/5 of terminal segment. Third segment with setule along anterior to distal margin. Terminal segment with claw on distal margin. L ratio between four segments 4: 1.6: 1: 1.1.

***L6*** (Fig. 6B). Four-segmented. First segment with one plumose seta antero-medially, not reaching end of the same segment, one plumose seta antero-distally reaching 1/3 of second segment. Second segment with setulae along anterior to distal margin, one bare seta antero-distally, reaching 1/4 of terminal segment. Third segment with setulae along anterior to distal margin. Terminal segment with claw-like seta on distal margin. L ratio between four segments 2.9: 1.7: 1: 1.08.

***L7*** (Fig. 6D). Four-segmented. First segment with one plumose seta antero-distally reaching 1/7 of the second segment. Second segment with setulae along anterior to distal margin, one plumose seta antero-distally reaching end of third segment. Third segment with setulae along anterior to distal margin. Terminal segment with one claw like seta on distal margin. L ratio between four segments 2.8: 2.6: 1: 1.36.

***GF*** (Fig. 6C). Basal part subtriangular. Ovary kidney-bean shaped, situated at the middle of the basal capsule. One caudal ramus seta present. One end of the body seta present.

###### Description of male.

***A1*** (Fig. 5C). Six-segmented. Fourth and penultimate segment fused. First segment without any seta. Second segment with setulae postero-medially and along anterior to distal margin. Third segment with setulae antero-proximally, one bare seta antero-distally, reaching end of fourth segment. Fourth segment with two bare setae antero-distally, one reaching end of terminal segment, another reaching half of same segment, one bare seta postero-distally, reaching end of terminal segment. Fifth segment with two bare setae antero-distally almost twice as long as terminal segment. Terminal segment with three bare setae on distal margin almost 2.5 times as long as same segment. L ratio between five segments 3.25: 6.5: 2.25: 2.42 (fused segment): 1.

***A2*** (Fig. 5B). Five-segmented. Exopod transformed into three-segmented spinneret seta. First segment without any seta. Second segment with two setae postero-distally, one plumose seta reaching slightly over half of terminal segment. Third segment with one bare seta postero-distally not reaching half of the terminal segment, two bare setae situated medio-distally, not reaching half of the terminal segment. Fourth segment with setulae along antero-distal margin; one bare seta on posterior-distal margin, reaching slightly over distal end of the same segment; one bare seta antero-medially, not reaching distal end of the same segment. Terminal segment with one claw and one short seta fused with it. L ratio between three segments (excluding terminal segment) 2.5: 1.1: 1: 1.3.

***Hp*** (Fig. 5D). Basal part subrectangular form with two bare setae on anterior medially. Distal lobe subtriangular with slightly cuneiform distal tip. Same lobe also vertically subdivided.

Other appendages same as in female.

### Key to living Paradoxostomatidae genera

**Table:** 

1	Md-palp transformed into whip-like seta	***Paracytherois* Müller, 1894**
–	Md-palp with distinct segments	**2**
2	Md-coxa styliform	**3**
–	Md-coxa with distinct teeth	**16**
3	Terminal claw on A2 as well as claws on all walking legs very short and hook-shaped	**4**
–	Terminal claw on A2 as well as claws on all walking legs not so short and hook-shaped	**6**
4	Mxl with only one endite	***Asterositus* Tanaka & Arai, 2017**
–	Mxl with two prominent endites	**5**
5	Terminal segment of A2 reduced (i.e. completely fused with terminal claw)	***Echinophilus* Schornikov, 1973**
–	Terminal segment of A2 not reduced (i.e. there is a clear division between the segment and the claw)	***Echinositus* Schornikov, 1973**
6	Terminal segment of A2 with 2 claws	**7**
–	Terminal segment with one claw	**10**
7	Hinge lophodont	***Boreostoma* Schornikov, 1993**
–	Hinge adont	**8**
8	Carapace with a postero-ventral spinula	***Calcarostoma* Schornikov & Keyser, 2004**
–	No postero-ventral spinula present	**9**
9	Mxl palp completely absent	***Lanceostoma* Schornikov & Keyser, 2004**
–	Mxl palp reduced into a seta	***Paradoxostoma* Fischer, 1855**
10	Hinge adont	**11**
–	Hinge lophodont	**13**
11	Posterior end of carapace with extension situated slightly above middle, anterior margin cuneiform	***Austroparadoxostoma* Hartmann, 1979**
–	Both anterior and posterior margins rounded	**12**
12	Mxl palp reduced into a medium size seta	***Pontostoma* Schornikov & Keyser, 2004**
–	Mxl palp absent	***Brunneostoma* Schornikov, 1993**
13	Terminal segment of A2 carrying a seta, at least half as long as the claw	***Obesostoma* Schornikov, 1993**
–	If present, seta is tiny	**14**
14	First endite on the Mxl at least ½ as long as the other two	***Bradystoma* Schornikov & Keyser, 2004**
–	First endite on the Mxl much shorter	**15**
15	Anterior margin of the carapace cuneiform, and antero-ventral surface flattened	***Acetabulastoma* Schornikov, 1970**
–	Anterior margin of the carapace rounded and antero-ventral surface not flattened	***Triangulastoma* Schornikov & Keyser, 2004**
16	Carapace with sieve-pores present	**17**
–	No sieve-pores present	**18**
17	Terminal segment of Md-palp with a strong claw	***Redekea* de Vos, 1953**
–	Terminal segment of Md-palp without a claw	***Chelonocytherois* Tanaka & Hayashi, 2019**
18	A2 with two strong terminal claws	***Flabellicytherois* Schornikov, 1993**
–	A2 with one terminal claw	**19**
19	L5 with claw-like postero-distal seta, and A2 not sexually dimorphic	***Violacytherois* Schornikov, 1993**
–	L5 with seta-like postero-distal seta, A2 sexually dimorphic	***Cytherois* Müller, 1884**

### Key to East Asian species of Cytheroisinae

**Table:** 

1	Carapace with sieve-pores present	***Chelonocytherois omutai* Tanaka & Hayashi, 2019**
–	Carapace without sieve-pores	**2**
2	Terminal segment of A2 with 2 claws	***Flabellicytherois bingoensis* (Okubo, 1990)**
–	Terminal segment of A2 with one claw and at the most 1 seta	**3**
3	L5 with claw-like postero-distal seta	**4**
–	L5 with seta-like postero-distal seta	**5**
4	A1 5-segmented (4^th^ and 5^th^ segments fused)	***Violacytherois sargassicola* (Hiruta, 1976)**
–	A1 6-segmented	***Violacytherois violacea* (Schornikov, 1974) and *V. flavoviolacea* Schornikov, 1993**
5	Terminal segment of L7 beside a claw carrying one additional seta (clearly visible)	**6**
–	Terminal segment of L7 carrying only one claw	**7**
6	A1 5-segmented (4^th^ and 5^th^ segments fused)	***Cytherois megapoda* Schornikov, 1993**
–	A1 6-segmented	***Cytherois gajinensis* sp. nov.**
7	Dorsal margin of the carapace highly arched	***Cytheoris decorata* Okubo, 1980**
–	Carapace more elliptical in lateral view	**8**
8	Fourth and 5^th^A1 segments lacking any seta posteriorly (but carrying 2 setae each anteriorly)	***Cytherois ikeyai* Nakao & Tsukagoshi, 2002**
–	Fourth and 5^th^A1 segments carrying one seta each posteriorly (in addition to 2 setae each anteriorly)	***Cytherois zosterae* Schornikov, 1975**


## Discussion

With the addition of *Cytherois gajinensis* there have been eleven *Cytherois* species described from East Asia, half of which are known from the shell only. Nevertheless, the shell shape of the new species is distinctly different from the fossil/subfossil ones. In addition, one of the subfossil species, *C. asamushiensis* from Aomori Bay in Japan (Ishizaki 1971), has been transferred to *Paracytheroma* Juday, 1907 by Schornikov (2006). Although the above key to species does not consider sexual characters, in order to avoid misidentification in a case that only one sex is available for study, the largest differences between not only East Asian but all living *Cytherois* species are in the morphology of the hemipenis. Additionally, the species differ in the presence of a sucker-type seta on the penultimate segment of the male A2. Among the East Asian species, only *C. ikeyai* seems to possess a seta (Nakao and Tsukagoshi 2002) like *C. gajinensis* does. The second antenna is sexually dimorphic in this genus, but this dimorphism in most of the species is related to the length of the penultimate segment in relation to other segments, and in females it is longer than in males. Of all other representatives of the genus that have the second antenna described/illustrated, males of the following species have a brush-like seta on the A2: *C. australis* Hartmann, 1989; *C. lignicola* Maddocks & Steineck, 1987; *C. vitrea* (Sars, 1866); and *C. neogracilis* Hartman & Peterson, 1985 (see Sars 1866; Hartmann 1964, 1989; Maddocks and Steineck 1987). It has to be pointed out that in these species the morphology of the transformed seta is quite different from the sucker-type seta found in the new species and *C. ikeyai*, and also its position is not on the penultimate segment (4^th^), but rather on the third. This, with the discrepancies in the number of claws on the terminal segment of A2, with few species having two instead of one (for example, *C. neogracilis*), suggests that the genus should be revised with the purpose of clarifying phylogenetic relationships between species.

The second species reported here, *Violacytherois sargassicola*, seems to be relatively widely distributed in East Asia, since it has been reported from Hokkaido (Hiruta 1976), Peter the Great Bay in Russia (Schornikov 1993), and Korea. There are no differences between the Korean and the other two records. *Violacytherois sargassicola* is morphologically very similar (both carapace and soft body parts) to *V. violacea* and *V. flavoviolacea*. Beside minute differences in the morphology of the hemipenis, the species mainly differ in the number of A1 segments. This needs to be taken with caution, because the division between segments can sometimes be obscure or partial. In the above key to species, *V. violacea* and *V. flavoviolacea* could not be distinguished based on their descriptions/illustrations (Schornikov 1974, 1993), and it is likely that the latter is junior synonym of *V. violacea*.

The following three genera currently included in the family Paradoxostomatidae (see Brandão et al. 2020) are not part of the above key, because they are known only after the carapace morphology: *Caribbella* Teeter, 1975, *Glyphidocythere* Ayress, Correge & Whatley, 1993, and *Neopellucistoma* Ikeya & Hanai, 1982. We also excluded *Nodoconcha* Hartmann, 1989, *Paracythere* Müller, 1894, and *Pseudeucythere* Hartmann, 1989. In contrary to all other Paradoxostomatidae, those genera have much more robust A1, stronger mandibular coxa, robust Md-palp and well-developed Mxl-palp. In fact, Hartmann (1989) placed both of his genera in *incertae sedis* cytheroids and they have been included in WoRMS database erroneously. Müller (1894) considered *Paracythere* a member of Cytheridae, while Martin and Davis (2001) placed it into Paradoxostomatidae. Despite our attempt to provide a key to Paradoxostomatidae, it has to be used with caution as many of the genera are in need of revision. We based our key on the most typical representatives of each genus although large genera (such as *Paradoxostoma* and *Cytherois*) include species that are morphologically, and thus probably also phylogenetically, very distinct.

## Supplementary Material

XML Treatment for Cytherois gajinensis

XML Treatment for Violacytherois sargassicola
